# The acoustic phase resonances and surface waves supported by a compound rigid grating

**DOI:** 10.1038/s41598-018-29149-4

**Published:** 2018-07-16

**Authors:** Joseph G. Beadle, Timothy Starkey, Joseph A. Dockrey, J. Roy Sambles, Alastair P. Hibbins

**Affiliations:** Electromagnetic and Acoustic Materials Group, Department of Physics and Astronomy, Physics Buidling, Stocker Road, University of Exeter, Exeter, EX4 4QL UK

## Abstract

We study the radiative and bound acoustic modes supported by a rigid grating formed of three same-depth, narrow grooves per unit cell. One of the grooves is twice the width of the other two, forming a ‘compound’ grating. The structure supports so-called ‘phase’ resonances where the phase difference of the pressure field between the grooves on resonance varies by multiples of π. We explore the dispersion of these modes experimentally by monitoring the specularly reflected signal as a function of the angle of incidence. In addition, by near-field excitation, the dispersion of the non-radiative surface modes has been characterised. Our results are compared with the predictions of a finite element method model.

## Introduction

Patterning of surfaces to control sound attenuation has been a topic of many studies. These include structuring surfaces to manipulate acoustic surface waves (ASWs)^1–3^ leading to increased transmission^4^, scattering from arrays of elastic scatterers to create sonic crystals to attenuate transmission^5–7^, as well as controlling the propagation of the wave using labyrinthine structures^8,9^. Recently, a number of works^10–13^ have shown that enhanced acoustic transmission of sound through sub-wavelength perforations (holes or grooves) can be achieved. These studies are somewhat analogous to the extraordinary optical transmission found in the electromagnetic domain explained by coupled surface wave and evanescent diffraction phenomena^14^. Work by Skigin and coworkers^11,15^ has shown that transmission of electromagnetic radiation through a so-called ‘compound grating’, comprising of more than one groove per unit cell, is significantly different to that for a simple groove grating. The additional complexity of the unit cell typically broadens the exisiting resonant mode (due to increased radiaitive and non-radiaitive losses), while a new, narrow (i.e., high-Q-factor) ‘phase’ resonant mode is observed. These phase resonances are characterised by the resonant acoustic fields in adjacent grooves varying by mulitples of π with strong field enhancement on resonance^15^.

Analogous behaviour in the acoustic domain was predicted by Wang *et al*.^13^ and then experimentally verified by Ward *et al*.^16^ who demonstrated phase resonances in compound-groove-gratings with different structure factors. Narrow resonant dips within the band of the broad transmission maxima were observed and attributed to evanescent diffractive coupling between adjacent cavities modes. More recently, Zhang *et al*.^17^ investigated the acoustic transmission for compound gratings comprising different square and triangular shaped elements; they reported some degree of control of the resonance frequencies.

In addition to transmission-type gratings, similar phase-resonance effects in reflection compound gratings have also been studied in the electromagnetic domain^18–20^. In a study by Fantino *et al*.^18^, a number of different metallic compound gratings were numerically investigated. For a transverse-magnetic-polarised incident beam, phase resonances were observed as maxima in the reflectivity spectrum of the surface, with strongly enhanced fields within the grooves. A similar phenomenon for reflection gratings has yet to be recorded in the acoustic domain.

**Figure 1:**
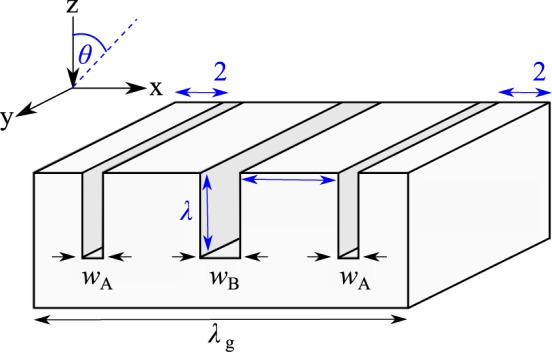
Schematic of a unit cell used in the experiment, comprised of three grooves per period (${\lambda }_{{\rm{g}}}$ = 19 mm) where the central groove is twice the width of the adjacent two. Here, *w*_A_ = 1 mm, *w*_B_ = 2 mm, *l* = *d* = 5 mm, and $\theta$ is the polar angle of incidence.

In this work, we explore fully the dispersion of the acoustic surface modes supported by a compound grating with three grooves per unit cell of two different widths (all of the same depth), see Fig. 1. This configuration was chosen as it allows for near critical coupling when there is perfect destructive interference between the reflected field and the internal field - the radiative and internal losses from the surface are balanced allowing for maximum absorption on resonance^21,22^. To determine the mode dispersion in the radiative region we record the reflectivity of the sample as a function of the polar angle of incidence, *θ*. In addition, in order to characterise the surface mode dispersion in the non-radiative region, we probe the propagation of trapped sound across the surface via near-field excitation and detection. Both the radiative and non-radiative features are attributed to Acoustic Surface Waves (ASWs), which are strongly dispersive in frequency-wavevector space, particularly on approach to 1^st^ Brillouin zone boundary $\,\frac{{k}_{{\rm{g}}}}{2},$ where ${k}_{{\rm{g}}}=\frac{2{\rm{\pi }}}{{\lambda }_{{\rm{g}}}}$ is the grating vector. Further the addition of additional grooves to the unit cell allows for surface modes to be supported beyond the first Brillouin zone^16^, which are scattered by integer multiples of ${k}_{{\rm{g}}}$ back into the first Brillouin zone to become features in the reflected signal.

Using the two experimental methods previously described we determine the dispersion of both the lowest order radiative branches and the non-radiative acoustic surface mode. The results are compared to the predictions of a finite element method (FEM) model. To the authors knowledge, this is the first observation of ASWs on a compound grating.

## Results and Discussion

Sound incident onto a flat rigid surface (i.e., acoustically-rigid, so no penetration of sound into the material is allowed) will have a reflectivity of unity. This will still be true if the sample is periodically structured, in the absence of loss and for frequencies below the onset of diffraction. However, this is not realistic because losses occur at a fluid-solid boundary due to the presence of thermal and viscous boundary layers^23^. Thermal losses arise because temperature gradients in the fluid irreversibly transfer heat into the walls. Viscous losses arise both in the bulk of the air but primarily in a thin boundary layer due to the no-slip condition at the wall causing a velocity gradient and thereby viscous dissipation. Associated with these viscous and thermal boundary layer effects are two boundary layer thicknesses^23^.

**Figure 2:**
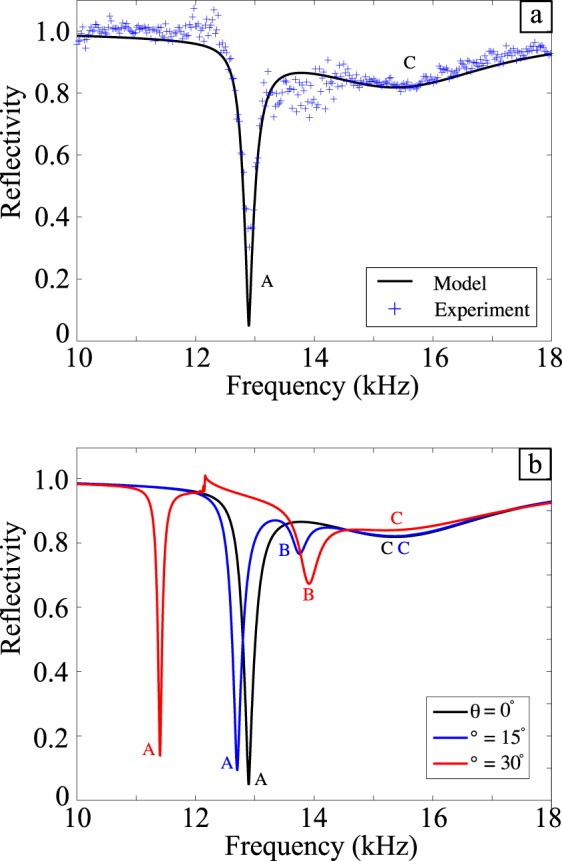
(**a**) Experimental reflectivity data for near-normal incidence (blue crosses) compared with the FEM model (solid line), model parameters are found in the Supplementary Material. (**b**) FEM model predictions of the reflectivity showing the reflectivity spectrum for different angles of incidences. The sharp feature at ~12 kHz for $\theta \,$ = 30° corresponds to the onset of diffraction where the in-plane component of the incident radiation *λ*_0×_ is comparable to *λ*_g_. As this condition is met, radiation is diffracted into unwanted loss channels rather than coupling to the surface mode.

The reflectivity as a function of frequency is shown in Fig. 2(a). The rather broad and shallow mode at 15.5 kHz (C) corresponds to a resonance where all the fields in the three grooves in a unit cell are in-phase. The fundamental mode of the grooves is the quarter-wavelength (*λ*/4) condition plus an end correction. The dependence of the modes’ resonant frequencies on the angle of incidence is shown in Fig. 2(b): off-normal excitation also reveals a third mode (B) that cannot be excited at normal incidence, to which there is increased coupling strength with increasing angle of incidence (*θ*). Also of interest is the angle dependence of the resonance frequency of mode (A) that shows a decrease in frequency as the angle of incidence is increased.

**Figure 3:**
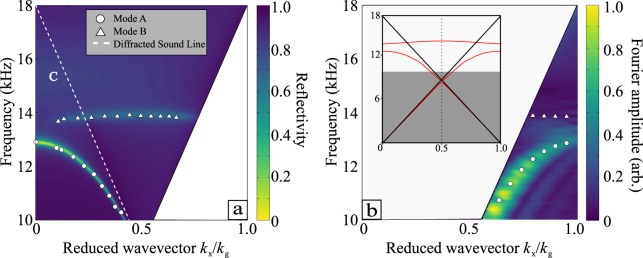
(**a**) Radiative domain in blue - frequency of reflectivity minima from experimental measurements (symbols) compared with predictions of the reflected intensity from the FEM model (colour-scale). The broad and shallow mode C has also been labelled for completeness. (**b**) Non-radiative region in blue - Fourier transform of the spatial near-field maps (colour-scale) compared with the predictions of the surface wave eigenmodes from the FEM model (symbols). Inset: Predictions of the dispersion obtained from the FEM model across a broader range of frequencies. The surface eigenmodes are shown in red, the solid black lines represent the sound line and onset of diffraction, and the shaded area represents frequency below our measured range (i.e., only the unshaded region of wavevector-frequency space is depicted in the main part of this figure).

From such data one obtains a mapping of much of the dispersion curve in the radiative region (i.e., for in-plane wavevector *k*_x_ < 2π*f*/*v*, where *f* is frequency and *v* is the speed of sound) for the plane containing the grating wavevector *k*_g_. Fig. 3(a) shows the experimental data obtained for the reflectivity measurements demonstrating the dispersion of the three modes, compared to the predictions of the reflectivity from a Finite Element Method (FEM) model^24^.

**Figure 4:**
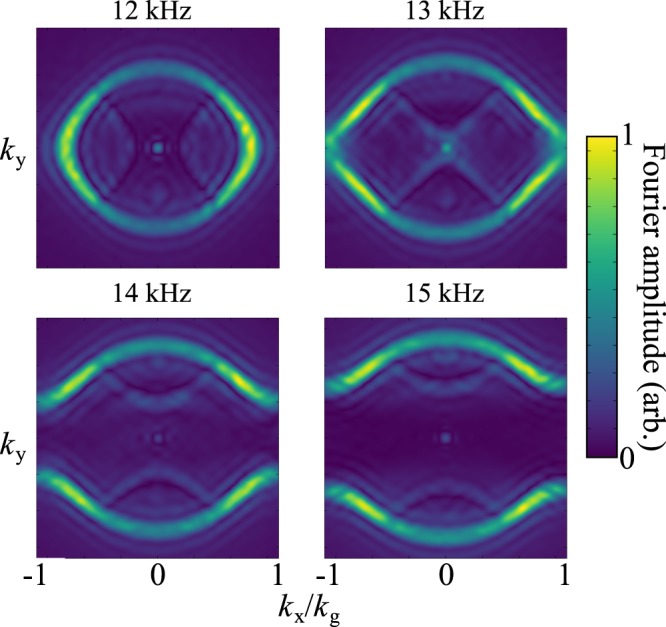
Experimental equifrequency contours within the first Brillouin zone (in *k*_x_) for 12, 13, 14 and 15 kHz. There is no periodicity in y, but the *k*_y_ axis is plotted on the same scale as *k*_x_.

We also explored the excitation of the bound surface modes supported by the sample, i.e., the dispersion of the modes *k*_x_ > 2π*f*/*v*, i.e., beyond the sound line. Fig. 4 demonstrates that the surface mode dispersion is close to being isotropic at the lowest studied frequencies, but becomes highly anisotropic as the frequency rises and the mode approaches the Brillouin zone boundaries.

From a composite of each of the isofrequency maps such as shown in Fig. 4, Fourier-transformed field measurement in any direction in k-space can be extracted to yield a representation of the mode’s dispersion. This is shown for the plane containing the grating vector in Fig. 3(b), where the experimental data is the colour scale and the points are the eigenvalues predicted by the FEM modelling. Note here the weak modulation of the intensity of the experimental signal along the dispersion curve: this arises from the finite size of the sample defining a limiting k-space resolution.

With three grooves per unit cell there are three degrees of freedom available. This leads to three different eigenmodes, i.e., three resonant features in reflection. The first is the broad and shallow branch (C) for which the field in each of the three grooves has the same phase in any given unit cell. Its frequency is approximately given by its wavelength being four times the groove depth, *d* - the fundamental resonance of the groove. When cavities are excited on resonance, evanescent end-effects occur at the opening of each cavity. These near-fields couple the groove resonances together over the surface in the form of a wave. For an ASW mode within the first Brillouin zone (BZ) only one pressure antinode per unit cell is allowed. This mode can be excited in the case of a monograting because the ASWs wavelength (*λ*_x_) approaches that of twice the grating wavelength (*λ*_g_): with one resonator per unit cell as the condition of one antinode per unit cell is satisfied. In the case of shorter ASW wavelengths (in the second BZ) two antinodes are required per unit cell with one being required over the rigid surface, this cannot occur. With the addition of an additional degree of freedom, i.e. a second groove per unit cell, the condition of two antinodes per unit cell may now be met. Then, by the process of first order diffraction this mode is observed in the radiative region of the first BZ. Extending the discussion to the case of the sample measured, with three resonators per unit cell a third mode existing within the third BZ can be excited. Hence, with the extra degree of freedom, ASWs with smaller wavelengths than *λ*_x_ = *λ*_g_ can now be excited as an eigenmode with three antinodes per unit cell is now available. Similar to the second mode, this mode is also scattered by diffraction into the radiative region of the first BZ. These modes can be seen in the inset of Fig. 3(b) as the two red lines in the radiative region.

**Figure 5:**
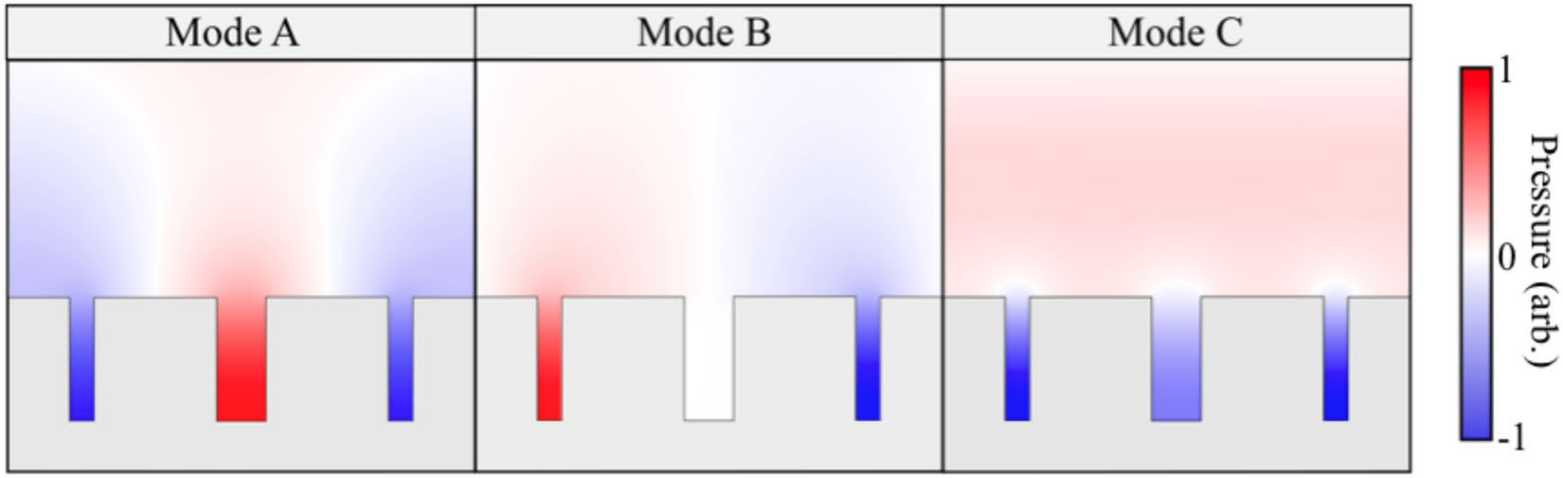
Pressure fields for phase resonances A (the central cavity fields being in antiphase with the outer two), B (fields in the outer two grooves being in antiphase, with the central one having zero amplitude), and C, the normal in-phase resonance. The scaling factors of the colour scale are 32.4, 21.7, and 3.5 respectively.

The acoustic field configurations for modes A, B and C are represented in Fig. 5. As evidence of the previous discussions, notice that the ASW wavelength *λ*_x_, matches the associated wavevector of the Brillouin zone boundary from which it was scattered; and note that mode C is a purely radiative mode and not confined to the surface. The relative pressure field in comparison to the nonresonant case, for modes A, B and C are 32.4, 21.7 and 3.5 respectively. When a comparison is made between the absorption and the relative pressure field strength, it becomes apparent that as the pressure field within the grooves increases the amount of absorption, seen as a reduction in reflectivity in Fig. 2, also increases.

The increased field on resonance is similar to the case of transverse-magnetic light incident on a metallic compound grating, however, the difference is that in the electromagnetic case the feature of the phase resonance gives a maximum in the reflectivity while in the acoustic case a minimum is observed. This is due to the relative backgrounds in the two cases: for p-polarised light, the phase resonance features as a sharp maximum in a low background, because of ^18^. For acoustic waves, the resonance is a sharp minimum in a high background.

From Fig. 3(a) it is apparent that mode B is not excited at normal incidence. This arises simply because the fields in the outer two cavities have to be in antiphase for this mode with the central cavity fields having zero amplitude at normal incidence. It is thus impossible to excite with a plane, normal incidence wave. Away from normal incidence there is a phase difference across a unit cell and this mode may now be excited. These phase resonances allow the possibility for developing narrow-band acoustic filters.

**Figure 6:**
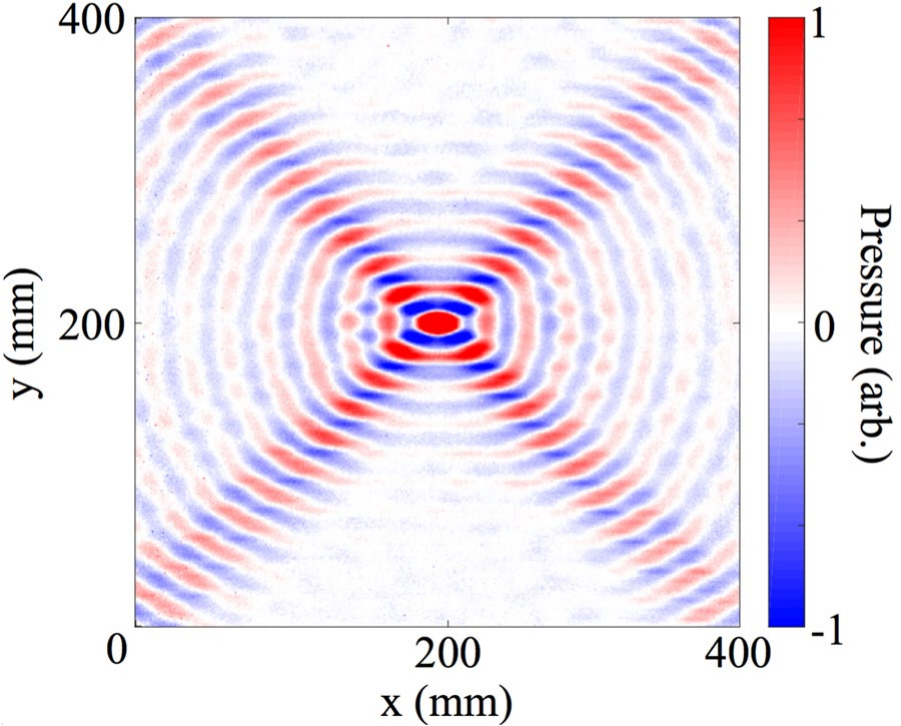
Experimental data for the instantaneous pressure fields at 13 kHz, showing that the power flow is strongly confined in four directions.

Note from Fig. 4 how the surface wave propagation becomes progressively more anisotropic as the frequency is increased. The equi-energy circle distorts first into an ellipse and then at frequencies above the first resonance of the system (at normal incidence) a band gap occurs where no mode is excitable in the x-direction and the equi-energy contour splits into curved lines. (The weaker features shown towards the centre of each image are modes scattered into the first BZ by first order diffraction, there are also reflections present due to the finite sample size). From these isofrequency contours the direction of the group velocity (***v***_g_) (determined by ***v***_g_ = ∇_k_*ω*, $\omega$ being angular frequency) is obtained and if it has a region which has constant gradient, acoustic beaming occurs where a range of wavevectors have the same ***v***_g_. An example of this effect in the frequency domain is shown in Fig. 6. Interestingly, for different frequencies the acoustic power is directed in different directions allowing for a frequency dependent directivity of acoustic power on the surface.

## Conclusions

In this study, a compound groove grating having three grooves per period which supports acoustic phase resonances and acoustic surface modes has been modelled and the results verified experimentally, showing a sharp minimum in the reflectivity spectrum, giving the possibility of a frequency specific acoustic filter. The surface was also found to support acoustic surface modes whose dispersion has been obtained and which, for a range of frequencies, exhibit frequency dependent directional acoustic power beaming.

## Method

### Non-radiative experiment

**Figure 7:**
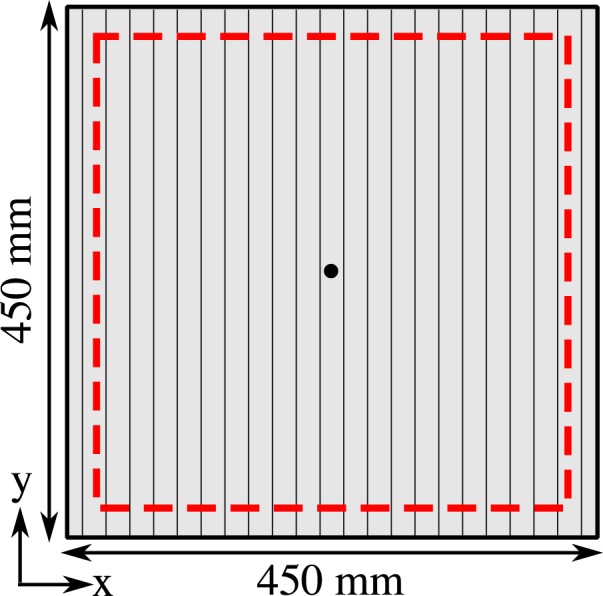
Schematic (not to scale) of the sample for the non-radiative experiment. The sample consists of 23 units where the cavities extend the whole y-length of the sample. Here, an acoustic pulse is emited through a hole in the centre of the sample (black dot) and the wave diffracts and couples to a surface wave. A microphone is then raster-scanned near the surface within the red dashed square recording the evolution of the wave as a function of time for every position.

In order to measure experimentally the non-radiative regime of the dispersion diagram, we position a loudspeaker behind the grating with the tip of an attached hollow cone positioned with its narrow tip inside a small hole of radius 1 mm drilled through the centre of the sample, a simple diagram can be seen in Fig. 7. The diffracted sound couples via high wave-vector components to the surface mode on the structured side. A microphone with a needle probe with its tip about 0.5 mm from the grating is then raster-scanned over the structured face of the sample. For every microphone position, a pulse is emitted from the loundspeaker and subsequently detected by the microphone. By performing a temporal Fourier transform on the detected signal, the amplitude and phase is determined, hence obtaining a spatial field-map for each frequency component. A 2D Fast Fourier Transform is then performed on each of the spatial field maps to create an iso-frequency k-space plot of the modes supported. A 400 mm by 400 mm scan area was chosen as this allows for sufficient resolution in k-space.

### Radiative experiment

**Figure 8:**
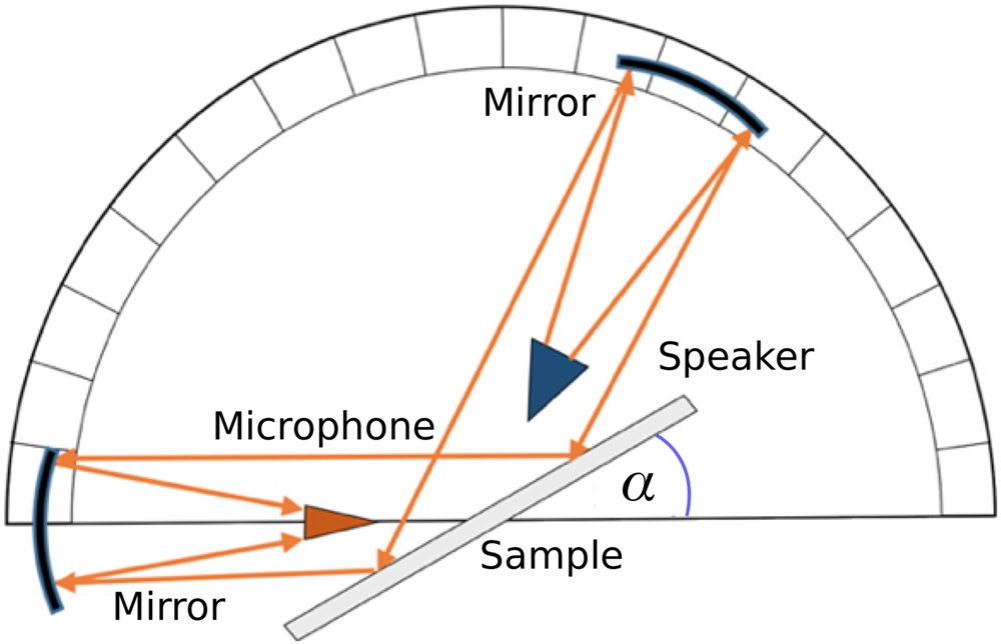
Schematic (not to scale) of the experimental setup, consisting of a speaker at the focus of a collimating mirror giving a plane wave incident onto the sample, the reflected signal being then focused by a second mirror onto the microphone. The speaker and microphone are placed below the central plane of the mirrors and sample such that they do not directly impede the acoustic beams. Here, *α* = 90*°* − *θ*.

In order to obtain the reflectivity from the grating in the radiative regime, a pulse-measurement technique in free space was used. A speaker is placed at the focus of a collimating mirror to provide a near-plane wave incident on the 450 mm–square sample, with a second collimating mirror used to re-focus the reflected signal onto the detecting microphone (Fig. 8). A Gaussian electrical pulse centred at 12 kHz was fed into the loudspeaker to provide a broad range of frequencies. A number of repeat measurements are recorded in the time-domain, and subsequently averaged. The time domain signals are then Fast Fourier Transformed (FFT) and the resulting frequency domain response is normalised to that of an unpatterned, rigid plate. In order to avoid this direct transmission between source and receiver three separate methods were used. Firstly, for near normal incidence (*θ* = 0°), the direct signal was removed simply by time-gating as the time between the two signals (directed and reflected) was large enough that there was no overlap. Secondly, for small angles (*θ* < 50°) a metal plate was inserted vertically between the source and receiver which proved to be sufficient to remove the direct signal. Finally, for the largest angles (*θ* > 50°), no plate was required since the placement of the speaker and the receiver meant that extremely little/no direct signal was measured. By moving the source, detector, and mirrors, the reflectance for incident angles (with the incident wavevector lying in the x-z plane) from near normal incidence (*θ* = 0°) to approximately 60° was measured.

A typical set of data recorded for near-normal incidence ($\theta \mathop{ < }\limits^{ \sim }0.1^\circ )$ is shown in Fig. 2(a): the strong minimum in reflectivity (A) is associated with the ‘phase-resonance’ mode^25^.

## Electronic supplementary material

Supplementary Material
