# Author Correction: The acoustic phase resonances and surface waves supported by a compound rigid grating

**DOI:** 10.1038/s41598-018-32261-0

**Published:** 2018-09-21

**Authors:** Joseph G. Beadle, Timothy Starkey, Joseph A. Dockrey, J. Roy Sambles, Alastair P. Hibbins

**Affiliations:** Electromagnetic and Acoustic Materials Group, Department of Physics and Astronomy, Physics Buidling, Stocker Road, University of Exeter, Exeter, EX4 4QL UK

Correction to: *Scientific Reports* 10.1038/s41598-018-29149-4, published online 16 July 2018

There are errors in Figures 1 and 2 of this Article.

In Figure 1 the symbol “l” has been omitted from 3 locations. In addition the symbol “λ” is incorrect and should read “d”.

**Figure 1:**
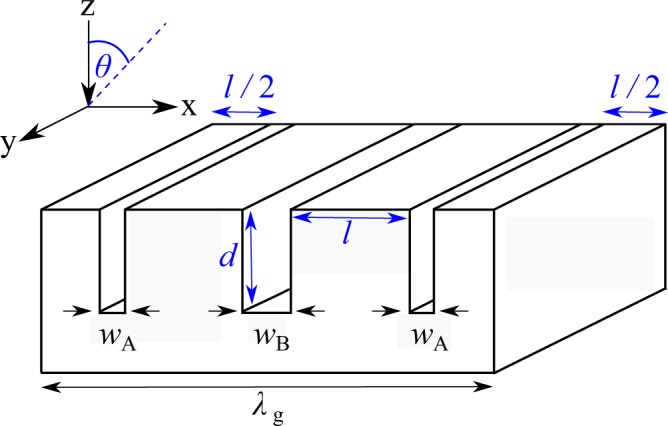
Schematic of a unit cell used in the experiment, comprised of three grooves per period (*λ*g = 19 mm) where the central groove is twice the width of the adjacent two. Here, *w*A = 1 mm, *w*B = 2 mm, *l* = *d* = 5 mm, and *θ* is the polar angle of incidence.

In Figure 2 the “θ” symbol has been omitted on the legend for 15° and 30°.

**Figure 2:**
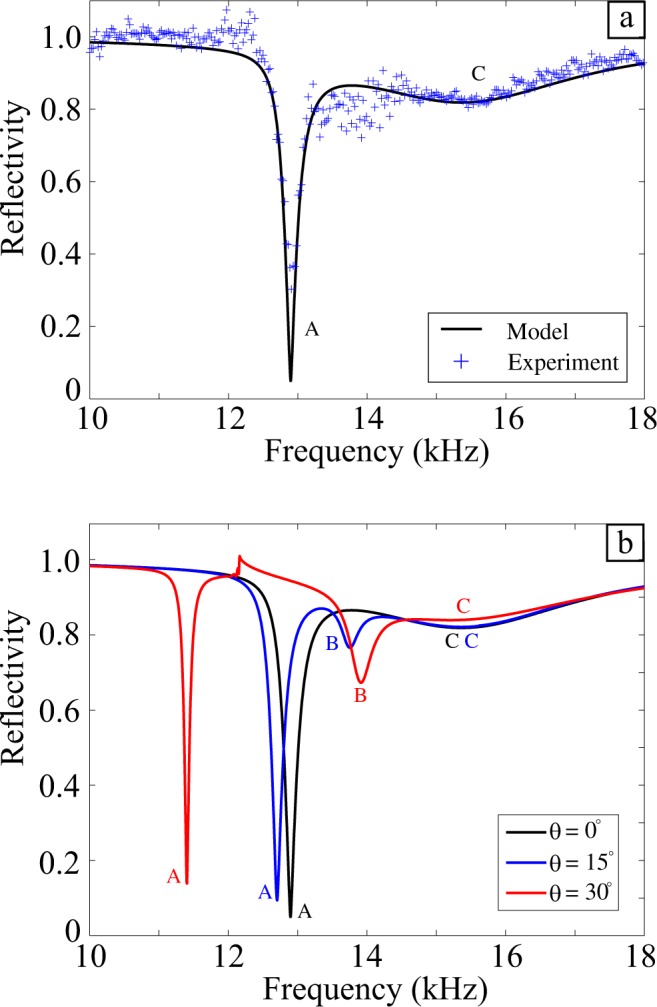
(**a**) Experimental reflectivity data for near-normal incidence (blue crosses) compared with the FEM model (solid line), model parameters are found in the Supplementary Material. (**b**) FEM model predictions of the reflectivity showing the reflectivity spectrum for different angles of incidences. The sharp feature at ~12 kHz for *θ* = 30° corresponds to the onset of diffraction where the in-plane component of the incident radiation *λ*_0×_ is comparable to *λ*g. As this condition is met, radiation is diffracted into unwanted loss channels rather than coupling to the surface mode.

