# Supplementary material for: The acoustic phase resonances and surface waves supported by a compound rigid grating
Source: Sci Rep. 2018 Jul 16;8:10701. doi: 10.1038/s41598-018-29149-4 (PMC6048070; doi:10.1038/s41598-018-29149-4)
Supplement: Supplementary file 1 — Supplementary Material [file 41598_2018_29149_MOESM1_ESM.docx]

Supplementary material for:

**The acoustic phase resonances and surface waves supported by a compound grating**

Joseph. G. Beadle*, Timothy. Starkey, Joseph. A. Dockrey, J. Roy. Sambles and Alastair. P. Hibbins

Electromagnetic and Acoustic Materials Group, Department of Physics and Astronomy, Physics Buildling, Stocker Road, University of Exeter, Exeter EX4 4QL UK

*Corresponding author: jgb206@exeter.ac.uk

**FEM modelling**

FEM modelling was performed using Comsol Multiphysics 5.2. Comsol’s thermoacoustic module and pressure acoustic module to were used capture fully the themo-viscous behaviour.

The velocity of sound in air was assumed to be 343.4 ms^-1^ at 20 ^o^C. Air was treated as an ideal gas, with the following physical parameters: density of air, $\rho_{0}=1.2754$ kg/m^3^; dynamic viscosity of air, *μ =*1.983 ${10}^{-5}$ Pa s; thermal conductivity, *k =* 0.0257 W/m K*;* and is the specific heat capacity of air, *C*_P_ = 1.005 kJ/kg.K.

To accurately model the losses associated with the boundary conditions imposed at the air/solid boundary interface an optimised boundary mesh was used.
